# Comparison of minor head trauma management in the emergency departments of a United States and Italian Children’s hospital

**DOI:** 10.1186/s13052-019-0615-0

**Published:** 2019-02-11

**Authors:** Brittany M. Stopa, Stefano Amoroso, Luca Ronfani, Elena Neri, Egidio Barbi, Lois K. Lee

**Affiliations:** 1 Computational Neuroscience Outcomes Center of Harvard, Brigham and Women’s Hospital, Boston, MA USA; 2 University of Trieste, Piazzale Europa, 1, 34127 Trieste, Italy; 3 Clinical Epidemiology and Public Health Research Unit, Institute for Maternal and Child Health IRCCS “Burlo Garofolo”, via dell’Istria 65/1, 34137 Trieste, Italy; 4 Division of Emergency Medicine, Boston Children’s Hospital, 300 Longwood Ave., Boston, MA 02115 USA

**Keywords:** Minor head trauma, Pediatric head trauma, Head trauma management, Clinical guidelines

## Abstract

**Background:**

Pediatric head trauma management varies between emergency departments globally. Here we aim to compare the pediatric minor head trauma management between a US and Italian hospital.

**Methods:**

We conducted a retrospective chart review of children 0–18 years old presenting after minor head trauma (Glasgow Coma Scale 14–15) from two emergency departments, in Boston, Massachusetts, United States and Trieste, Italy, between January and December 2013. Frequencies of demographic, clinical, and management characteristic were calculated. We compared rate ratios for characteristics of patients receiving cranial computed tomography (CT) scans between the two populations.

**Results:**

There were 1783 patients in Boston, Massachusetts and 183 patients in Trieste, Italy. Patients in Boston had more reported neurologic symptoms (61.2%) than in Trieste (6%) (*p* < 0.001). More CT scans were ordered on the patients in Boston (17.3% vs. 6.6%) (*p* < 0.001), while more children were hospitalized in Trieste (55.7% vs. 8.6%) (*p* < 0.001). Patients with neurological symptoms more commonly had a CT scan in Trieste (45.5%) than in Boston (23.5%) (RR 0.52, 95% CI 0.27, 1.00), while more patients without neurological symptoms had CTs in Boston (7.5%) than in Trieste (4.1%) (RR 1.85, 95% CI 0.86, 4.00). Assignment of triage levels and definitions of head injury severity varied considerably between the two hospitals, resulting in dissimilar populations presenting to the two hospitals, and thus, differences in the management of these children.

**Conclusion:**

The population of head trauma patients and the management of pediatric minor head trauma differs between Boston and Trieste, with a preference for CT scans in Boston and a preference for hospitalization in Trieste. Clinical guidelines used at each institution likely lead to this variation in care influenced by the different patient populations and institutional resources.

## Background

Head injury is a leading cause of emergency department (ED) visits for children in hospitals worldwide, and traumatic brain injury (TBI) is a leading cause of pediatric death and disability. When a pediatric patient presents with head trauma to the ED, the clinician has several strategies to use in the management of the patient, primarily observation or cranial computed tomography (CT) evaluation [1–3]. If there is concern for a potential clinically important traumatic brain injury (ciTBI), which is defined as traumatic brain injury requiring a neurosurgical procedure, hospitalization for > 2 nights or resulting in death, CT is the study of choice for evaluation as it is highly sensitive for detecting intracranial injury [4, 5].

CT use had been steadily increasing over the last decade and then plateaued [6–8]. Although cranial CT is the gold standard to identify cranial and intracranial lesions, it also exposes patients to ionizing radiation, which is associated with an increased risk of fatal malignancies [9, 10]. The effective use of cranial CT for diagnosing TBI must, therefore, be balanced with the potential malignancy risk to the patient and the cost of the exam.

To guide the clinician in the clinical decision-making in the evaluation of pediatric head trauma, several guidelines have been developed. Some of these include: the U.S. Pediatric Emergency Care Applied Research Network (PECARN), [11] the United Kingdom’s Children’s Head Injury Algorithm for the Prediction of Important Clinical Events (CHALICE), [12] and the Canadian Assessment of Tomography for Childhood Head Injury (CATCH) [13]. In Italy, the Italian Society of Pediatric Emergency Medicine (SIMEUP) and the Italian Society of Pediatrics (SIP) have drafted a national set of guidelines for the management of pediatric head injury [14]. As international consensus on pediatric head trauma management has not been reached, these guidelines are utilized by different hospitals and trauma care facilities internationally to aid in managing pediatric head trauma patients [15–19]. This may be due to differences in mechanisms of injury and severity of head trauma presenting to different hospitals, as well as different clinical preferences and hospital resources [19].

The objective of the present study is to compare the clinical management of head trauma in two pediatric hospitals, in the U.S and Italy. By comparing the management of head trauma between these two institutions, it may be possible to elucidate whether certain management guidelines are more effective than others and to develop a framework for international recommendations for the evaluation and management of pediatric head trauma.

## Methods

### Study design, setting, and participants

This study is a retrospective analysis of children and adolescents with head trauma presenting to two pediatric EDs, one in Boston, Massachusetts, U.S. and one in Trieste, Italy, between January 1, 2013 and December 31, 2013. Patient records were reviewed at Boston Children’s Hospital and the Institute for Maternal and Child Health, Istituto Di Ricovero e Cura a Carattere Scientifico (IRCCS) Burlo Garofolo, in Trieste, for patient treatment, management, and outcomes. Boston Children’s Hospital is a tertiary care referral hospital that evaluates over 60,000 patients in the ED annually, and includes a neurosurgery service available for emergent operations 24 h a day. The Institute for Maternal and Child Health IRCCS Burlo Garofolo is a tertiary care referral hospital with an annual ED volume of about 25,000. The Institute has no dedicated pediatric neurosurgery and the referral neurosurgery unit is located in the city general hospital, located at a three kilometers distance.

Ethics approval for this project was sought and granted by both hospitals. The institutional review board at each hospital approved the study protocol. For this type of study, formal consent is not required.

All children with minor head trauma and an initial Glasgow Coma Score (GCS) ≥14, who presented to the ED within 24 h of injury were included in the study population. In Boston patients < 18 years of age were included, and in Trieste children < 15 years of age were included, due to current local criteria for pediatric services. Children were excluded if the head injury was due to suspected non-accidental trauma (child abuse).

The triage categories and definitions of severity of head trauma varied by institution (Tables 1 and 2). In Boston, every child with head trauma, regardless of initial presentation and GCS score, is evaluated and managed at the hospital. A minor head trauma evidence-based guideline, based on the PECARN TBI clinical decision rule, is utilized in the clinical decision making regarding observation vs. CT for children with GCS ≥ 14 in Boston (Table 3). In contrast, children presenting with head trauma in Trieste primarily have GCS 14–15. Those patients with a GCS of ≤13 or those with altered mental status or loss of consciousness are considered major head trauma and routinely had cranial CT for evaluation. Patients in Trieste with a GCS ≤13 and abnormal CT findings from the traumatic head injury are referred to an external neurosurgery center. Patients with a GCS ≤ 8 are not evaluated in the Trieste pediatric ED, but are taken by ambulance directly to an external neurosurgery center. The clinical decision rule used in Trieste, an adapted PECARN algorithm, is summarized in Table 4.

**Table 1:** Differences in trauma triage categories

Head Trauma Triage Levels, Boston Children’s Hospital	
Level 1 (Most emergent)	Unresponsive or with depressed mental status after significant head trauma. Will need to go to OR or ICU immediately.
Level 2 (Emergent)	Altered mental status (may have repetitive questioning or be slow to respond), lethargic, but able to respond verbally.
Level 3 (Urgent)	Usually minor head trauma, may have vomiting and headache, but are awake and alert.
Level 4 (Least urgent/Non-urgent)	Minor head trauma, appear normal with no headache or vomiting. Usually these patients present after falling while running or after running into an object.
Head Trauma Triage Levels, Institute for Maternal and Child Health IRCCS Burlo Garofolo	
Red (Emergent)	One or more of: Critical vital parameters, Fracture of the base/exposed fracture, Penetrating wound or scalp, Severe dynamic^a^, Anisocory or pupils not reacting to light or gaze deviation, Coagulopathy, Infant with bulging fontanelle and weeping crying
Yellow (Intermediate)	One or more of: Prostrate child, GCS 13 or less, Loss of consciousness, Amnesia/syncope/dizziness, Persistent vomiting > 2 h from the head trauma, Persistent headache > 2 h or worsening headache, Post-traumatic convulsion resolved, Newborn, Depressed fracture, Scalp or face tear, Irritability or incessant crying, Diplopia, Soft swelling of the head
Green (Mild)	One or more of: 1–2 episodes of vomiting < 2 h from the head trauma, Headache at the impact point, GCS 14–15, Mild dynamic^a^, Infant > 6 months with no symptoms, Cephalohematoma
White (Trivial)	Head trauma with no signs or symptoms > 6 h

^a^Dynamic includes: Fall from height < 1 m, Impact against elastic or dampening surface, Fall from moving vehicle

**Table 2:** Definitions of severity of head trauma

Definitions of Severity of Head Trauma at Boston Children’s Hospital	
Minor Head Trauma	Characterized by all of the following: GCS 14–15, No fracture signs of the skull base, No focal neurologic deficit
Major Head Trauma	Characterized by one or more of the following: GCS ≤ 13, Skull base fracture signs, Depressive fracture of the cranial vault, Focal neurological deficits (sensory, motor, visual, verbal), Post-traumatic seizure
Severe Head Trauma	Major head trauma with a GCS ≤ 8 or rapid deterioration of the state of consciousness and must include the involvement of the anesthetist for airway management
Definitions of Severity of Head Trauma at Institute for Maternal and Child Health IRCCS Burlo Garofolo	
Minor Head Trauma	Patients with a GCS 14–15
Major Head Trauma	Patients with a GCS ≤ 13, including those with any altered mental status or loss of consciousness
Severe Head Trauma	Patient with a GCS ≤ 8 or rapid deterioration of the state of consciousness and need for an anesthetist for airway management.

**Table 3:** Summary of the Head Trauma EBG Algorithm© used in the Emergency Department at Boston Children’s Hospital^a^

Age ≥ 2	
Head CT recommended if:	Altered mental status (GCS of 14, agitation, sleepiness, slow response or repetitive questioning) Clinical signs of basilar skull fracture 3 of more of the following predictors are present: Loss of consciousness, history of vomiting, severe injury mechanism, severe headache
ED observation recommended if:	1–2 of the following predictors are present: Loss of consciousness, history of vomiting, severe injury mechanism, severe headache
Age < 2	
Head CT recommended if:	Altered mental status (GCS of 14, agitation, sleepiness, slow response or repetitive questioning) Palpable skull fracture 3 of more of the following predictors are present: Non-frontal scalp hematoma, loss of consciousness > 5 s including post-traumatic seizures, severe injury mechanism, acting abnormally per parent
ED observation recommended if:	1–2 of the following predictors are present: Non-frontal scalp hematoma, loss of consciousness > 5 s including post-traumatic seizures, severe injury mechanism, acting abnormally per parent

^a^See full algorithm in Nigrovic et al. [20]

**Table 4:** Adapted PECARN Algorithm for Management of Children with Minor Head Trauma at Institute for Maternal and Child Health IRCCS Burlo Garofolo^a^

High-risk	Patients with at least one of the following: GCS ≤ 13 or drop of 2 points since arrival, focal neurologic signs, loss of consciousness > 5 min, signs of basal or complicated skull fracture.
	CT recommended.
Moderate-risk	Patients who do not present any of the above reported features and whose risk of intracranial injury and subsequent management (CT or observation alone) is differentiated according to the presence of specific clinical predictors or the combination given by the severity of trauma mechanism with the presence and site of large scalp hematoma (the last feature considered only for children < 2 years).
	CT is recommended if:
	For children < 2 years amnesia is introduced alongside the other original predictors and, in the presence of isolated vomiting, CT is suggested if there was repetitive vomiting (more than 4 episodes) or persistent vomiting for more than 6 h after head trauma and a negative personal history for recurrent vomiting or motion sickness.
	Observation in the ED is recommended if:
	The recommended duration of observation in the ED for patients who did not undergo a CT is at least 6 h for trauma and at least 12 h for infants < 6 months.
Low-risk	Patients with absence of any of the features of the high- and moderate-risk groups and the possible presence of up to 4 episodes of vomiting immediately after trauma, mild headache confined to site of trauma, or loss of consciousness of only a few seconds.
	No imaging recommended.

^a^Modified from Bressan et al. [21]

### Data collection

Patient medical record databases at each hospital were queried from January 1, 2013 through December 31, 2013 for head injury by International Classification of Diseases-9th Edition-Clinical Modification (ICD-9-CM) codes (800–804.9, 850.0–854.0, 959.0).

Boston used an electronic medical record during this time, and Trieste used paper records. From those selected patient records, the following data points were manually extracted from the medical records: age, sex, neurological symptoms (headache, vomiting, lethargy, seizure, loss of consciousness, altered mental status, not acting normally per parents or focal neurologic exam), if CT was performed, CT findings (if applicable), disposition (discharged home, hospital admission), if neurological procedure was performed (Boston only), and final diagnosis.

### Outcome measures

The primary outcomes are related to management of pediatric head trauma patients as measured by CT use and disposition from the ED.

### Statistical analysis

Descriptive frequencies were calculated to summarize the populations at the two participating hospitals. Continuous variables were reported as medians and interquartile ranges (IQR) and categorical data as numbers and percentages. The two populations were compared with the Pearson Chi-Square test or Fisher exact test when appropriate for categorical variables and with the non-parametric Mann Whitney U test for continuous variables, as a non-normal distribution of data was present. Rate ratios (RR) with 95% confidence intervals (CI) were calculated to compare the differences in CT use between Boston and Trieste based on presence or absence of neurological symptoms. A two-sided *p*-value < 0.05 was considered statistically significant.

## Results

During the one-year study period, there were 1966 patients in the total study sample who were evaluated for minor head trauma: 1783 children in Boston and 183 children in Trieste. In both hospitals, the majority of patients with head trauma were male. In Trieste, the median age was higher (7.0 years) compared to Boston (5.0 years) (*p* = 0.007) (Table 5). Neurologic symptoms, as locally defined, were present in 61.2% of Boston’s patients (1092/1783) and 6% of Trieste’s patients (11/183) at the time of presentation to the ED (*p* < 0.001). Cranial CT was performed on 309 patients in Boston (17.3%) and 12 patients in Trieste (6.6%) (*p* < 0.001). Among those with cranial CT in Boston, 158 (51%) had the CT performed at the referring institution, prior to coming to Boston Children’s Hospital. Among those seen initially in Boston (not transferred from a referring institution), only 151 (10%) had a CT evaluation for their head trauma. In Boston 154 patients were hospitalized (8.6%), and in Trieste 102 patients were hospitalized (55.7%) (*p* < 0.001).

**Table 5:** Study population demographic and clinical characteristics, GCS 14–15

Characteristics	Boston (*n* = 1783)	Trieste (*n* = 183)	*p*
Age Group, median (IQR)	5.0 (1.0–12.0)	7.0 (2.0–13.0)	0.007
Sex, number, n (%)^c^			
Male	1121 (62.9%)	112 (61.2%)	0.65
Female	661 (37.1%)	71 (38.8%)	
Neurologic symptoms, n (%)			
No	691 (38.8%)	172 (94%)	< 0.001
Yes	1092 (61.2%)	11 (6.0%)	
Neurologic symptoms, n (%)			
Headache^a^	653 (59.8%)	6 (54.5%)	–
Vomiting^a^	292 (26.7%)	4 (36.3%)	
Lethargy^a^	184 (16.8%)	2 (18.1%)	
Seizure^a^	29 (2.7%)	1 (9.1%)	
Loss of consciousness^a^	164 (15.0%)	2 (18.1%)	
Altered mental status^a^	172 (15.8%)	0 (9.1%)	
Not acting normally, per parents^a^	240 (22.0%)	1 (9.1%)	
Focal abnormal neurologic exam^a^	24 (2.2%)	3 (27.2%)	
Cranial CT performed, (%)	309 (17.3%)	12 (6.6%)	< 0.001
Disposition, n (%)			
Admitted to the hospital	154 (8.6%)	102 (55.7%)	< 0.001
Neurosurgical procedure, n (%)	6	0	
Intracranial monitoring^b^	0		
Craniotomy^b^	4 (66.7%)		–
Epidural evacuation^b^	4 (66.7%)		
Skull fracture repair^b^	4 (66.7%)		

^a^percentages calculated on children with presence of neurologic symptoms

^b^percentages calculated on children with neurosurgical procedure

^c^1 missing data

We analyzed separately subjects with and without neurologic symptoms after minor head trauma. Inclusion criteria for neurologic symptoms were headache, vomiting, and lethargy. There were 1092 (61.2%) patients in Boston with neurologic symptoms and 11 (6.0%) in Trieste, according to local definition of neurologic symptoms. The median ages respectively were 9.0 years (IQR 3.0–14.0) and 12.0 years (IQR 5.0–16.0) (*p* = 0.22). In this group a CT scan was performed in 23.5% of Boston patients (257/1092) and in 45.5% of Trieste patients (5/11) (RR 0.52, 95% CI 0.27, 1.00) (Table 6).

**Table 6:** Characteristics of Patients with CT for Head Trauma Evaluation, GCS 14–15

	Boston *N* = 309	Trieste *N* = 12	Rate Ratio (95% CI)	*p*-value
Age				
Median (IQR)	7.0 (2.0–13.0)	6.5 (2.0–11.5)		0.60
< 2 years, n (%)	73 (23.6%)	2 (16.7%)	1.02 (0.97–1.06)	0.74^a^
≥ 2 years, n (%)	236 (76.4%)	10 (83.3%)		
Sex, n (%)				
Male	195 (63.3%)	8 (66.7%)	0.99 (0.95–1.04)	1.00^a^
Female	113 (36.7%)	4 (33.3%)		
Neurological Signs, n (%)				
Yes	257 (83.2%)	5 (41.7%)	1.11 (1.01–1.22)	0.002^a^
No	52 (16.8%)	7 (58.3%)		

^a^Fisher exact test

There were 691 patients with no neurologic symptoms in Boston and 172 in Trieste. The median ages respectively were 2.0 years (IQR 0.0–5.0) and 6.5 years (IQR 2.0–12.0) (*p* < 0.001). CT scan was performed in 52 subjects without neurological symptoms in Boston (7.5%) vs 7 (4.1%) in Trieste (RR 1.85, 95% CI 0.86, 4.00). Characteristics (age, sex, presence of neurological symptoms) of patients who had CT scans in the two institutions are described in Table 6.

## Discussion

This study comparing management of minor head trauma between a U.S. and Italian children’s hospital revealed substantial international differences, including the way patients are triaged for care between the two hospitals, the definitions in the triage level and severity of head trauma, and the use of CT for patients between the two institutions. As a result each institution then managed their respective minor head trauma populations differently. Overall, the use of cranial CT was higher in Boston compared to Trieste. However, more children were hospitalized for their head injury in Trieste compared to Boston.

The patient populations with head trauma as well as the assignment of triage levels and definitions of head injury severity varied considerably between the two hospitals, resulting in dissimilar populations presenting to the two hospitals and with differences in the management of these children. In Boston, children presenting with any severity of head trauma are evaluated and managed, including those requiring neurosurgery or intensive care unit (ICU) level of care. Whereas in Trieste, any patient at risk for a more severe head injury is transferred to another facility that has neurosurgical services available. The distribution of triage levels demonstrates a higher proportion of children with lower severity of symptoms presenting in Boston compared to Trieste. This most likely accounts for the significantly higher prevalence of neurological symptoms in the Boston children, compared to those in Trieste.

In Boston, the Pediatric Emergency Care Applied Research Network (PECARN) guidelines are employed to aid clinicians in deciding whether to order a head CT or observe the patient [11]. The Evidence Based Guidelines (EBG) algorithm© [20] (summarized in Table 3) summarizes these guidelines and recommends CT scan only if a patient has 3 or more PECARN risk factors. Children with two or fewer PECARN risk factors are recommended for observation for the development of any signs or symptoms of intracranial injury. In Trieste, an adapted PECARN guideline is employed to aid clinicians in their management decisions for minor head injury patients (Table 4), in accordance with national guidelines regarding pediatric head injury management [14].

The guidelines at both institutions provide clinicians with similar advice regarding observation for low-risk patients and CT use for intermediate-risk patients. Overall, there was a higher rate of CT use in Boston compared to Trieste. The difference in CT use between Boston and Trieste is partially attributable to Boston being the major referral facility for pediatric emergency care in the region. It is interesting to note that 51% of the CT scans for the Boston patients were done at referring hospitals prior to the patient arriving to the Boston Children’s Hospital ED. As Boston Children’ Hospital is one of the largest pediatric trauma centers in the region, many children with traumatic injuries are referred to this institution for more definitive management and care. For many of these patients, if they had been evaluated at Boston Children’s Hospital first, the PECARN guidelines may have recommended observation over CT scan, as only 10% of children who were evaluated initially at Boston Children’s Hospital had a CT performed. The difference in CT use between Boston and Trieste was not statistically significant when stratified by neurologic symptoms, although the sample size from Trieste was limited.

In contrast, the rate of admission for observation was significantly higher in Trieste compared to Boston, and these admissions were not for intensive neurosurgical monitoring or procedures. This demonstrates the differences in management preferences for minor pediatric head trauma between the institutions, which highlights some of the international differences in head trauma management. These global differences in CT rates are likely due to triage differences, institutional differences, and variations in the use of different decision rules. Even with a disparity in CT rates between countries, the effective impact on patient safety and outcomes is minimal. However, there are arguments to be made for the reduction of extraneous CT use, which include the radiation risk to patients [9, 10] and the financial cost incurred [22].

Several head injury decision rules have been developed: PECARN, [11] CATCH, [13] and CHALICE [12]. The most widely used of these is the PECARN rule, which is utilized in modified forms in Boston and Trieste. There have been studies conducted to compare the relative accuracies of these decision rules in various international settings, including Australia [18, 23, 24] and New Zealand [1]. In addition, there are also studies demonstrating that these decision rules can be successfully implemented in various international settings, including: the PECARN rule in Padova, Italy, [21] Nantes, France, [17] Tehran, Iran, [19] Boston, U.S., [25] and Tokyo, Japan [16].

In contrast, a cogent study to evaluate the suitability of implementing one of these rules in Denmark found that the limited availability of CT in Scandinavia would make it inappropriate to implement CHALICE or PECARN guidelines [5]. Thus, clinical decision rules for the management of head trauma may not be universally applicable to all institutions due to differences in institutional resources and patient population with varying mechanisms and severities of injury.

Our study has some limitations. As this was a retrospective database study, it is possible some patients were missed. However, a comprehensive list of ICD-9-CM codes was used to identify patients with head trauma from the electronic medical record in Boston. Some individual medical records may not have included all of the data elements included in the study. Also, between the two hospitals, some data elements were not consistently recorded, which limited some of the analyses. There is also likely some variation in the definition of some of the variables, including the neurologic symptoms, which may account for some of the differences in the population. The sample size between the two hospitals is also quite different with a small proportion of children having a cranial CT in Trieste, which limits interpretation of some of the analyses.

## Conclusion

In this study of pediatric minor head trauma patients in Boston and Trieste, we demonstrated variation in the triage of patients, definitions of triage and head trauma severity levels, CT use, and hospital admission. Both hospitals successfully implemented some form of the PECARN head trauma clinical decision rule as their guideline. Institution-specific and population-specific differences are important to consider when implementing these type of guidelines to provide optimal care with appropriate testing.

## Abbreviations

CATCH: Canadian Assessment of Tomography for Childhood Head Injury
CHALICE: Children’s Head Injury Algorithm for the Prediction of Important Clinical Events
CI: Confidence interval
ciTBI: Clinically important traumatic brain injury
CT: Computed tomography
EBG: Evidence based guideline
ED: Emergency department
GCS: Glasgow Coma Scale
ICD-9-CM: International Classification of Diseases-9th Edition-Clinical Modification
ICU: Intensive care unit
IQR: Interquartile range
IRCCS: Istituto Di Ricovero e Cura a Carattere Scientifico
PECARN: Pediatric Emergency Care Applied Research Network
RR: Rate ratio
SIMEUP: Italian Society of Pediatric Emergency Medicine
SIP: Italian Society of Pediatrics
TBI: Traumatic brain injury
U.S.: United States
